# Antioxidant, Cytotoxic, and Toxic Activities of Propolis from Two Native Bees in Brazil:* Scaptotrigona depilis* and* Melipona quadrifasciata anthidioides*

**DOI:** 10.1155/2017/1038153

**Published:** 2017-03-09

**Authors:** Thaliny Bonamigo, Jaqueline Ferreira Campos, Tamaeh Monteiro Alfredo, José Benedito Perrella Balestieri, Claudia Andrea Lima Cardoso, Edgar Julian Paredes-Gamero, Kely de Picoli Souza, Edson Lucas dos Santos

**Affiliations:** ^1^School of Environmental and Biological Science, Federal University of Grande Dourados, Dourados, MS, Brazil; ^2^Course of Chemistry, State University of Mato Grosso do Sul, Dourados, MS, Brazil; ^3^Department of Biochemistry, Federal University of São Paulo, SP, Brazil; ^4^Interdisciplinary Center of Biochemistry Investigation, University of Mogi das Cruzes, Mogi das Cruzes, SP, Brazil

## Abstract

Propolis is a natural mixture of compounds produced by various bee species, including stingless bees. This compound has been shown to exhibit antioxidant, antiproliferative, and antitumor activities. The present study aimed to determine the chemical constituents as well as the antioxidant, cytotoxic, and toxic activities of ethanol extracts of propolis obtained from the stingless bees* Scaptotrigona depilis* and* Melipona quadrifasciata anthidioides*, which are found in Brazil. Phytosterols, terpenes, phenolic compounds, and tocopherol were identified in the ethanol extracts of propolis (EEPs) in different concentrations. The compounds stigmasterol, taraxasterol, vanilic acid, caffeic acid, quercetin, luteolin, and apigenin were found only in EEP-M. The EEPs were able to scavenge the free radicals 2,2-diphenyl-1-picrylhydrazyl and 2,2′-azino-bis(3-ethylbenzothiazoline-6-sulfonic acid) and protected human erythrocytes against lipid peroxidation, with the latter effect being demonstrated by their antihemolytic activity and inhibition of malondialdehyde formation. The EEPs showed cytotoxic activity against erythroleukemic cells and necrosis was the main mechanism of death observed. In addition, the concentrations at which the EEPs were cytotoxic were not toxic against* Caenorhabditis elegans*. In this context, it is concluded that EEP-S and EEP-M show antioxidant and cytotoxic activities and are promising bioactive mixtures for the control of diseases associated with oxidative stress and tumor cell proliferation.

## 1. Introduction

Stingless bees, also known as meliponini, belong to the tribe Meliponini and are distributed across more than 32 genera [1]. Most species in this group exhibit eusocial habits and are found in tropical and subtropical regions, and 244 species have been described in Brazil [2, 3].

This group of bees plays an important ecological role, contributing to the preservation of plant species through pollination. Moreover, they produce pollen, honey, wax, and propolis, which are used in the hive and are consumed by humans as nutraceuticals [4–6]. Among these compounds, propolis is obtained through the collection of exudates from different parts of plants and combined with salivary enzymes from bees, resulting in a resinous material that is used to repair cracks and damage to the hive, defend against microorganisms, and mummify the bodies of other insects [7, 8].

Propolis is generally composed of 50% to 60% resins and balsams, 30% to 40% waxes, 5% to 10% essential oils, and 5% pollen grains and micronutrients, with small amounts of vitamins B1, B2, B6, C, and E [9]. The color and chemical composition of this resin vary depending on the plant species from which bees collect the raw material and the bee species that produces it [8].

Therefore, studies on propolis collected from different geographical regions and bee species are of great importance because these elements affect the chemical composition and, consequently, the biological properties of propolis.

The therapeutic activity of propolis from stingless bees has been widely investigated in recent decades, including descriptions of its antioxidant activity [10–12], antimicrobial activity [5, 13, 14], anti-inflammatory activity [15, 16], and antitumor activity [7, 12, 17].

Among bee species,* Scaptotrigona depilis*, popularly known as “mandaguari” and* Melipona quadrifasciata anthidioides*, known as “mandaçaia,” are stingless species found in South American countries including Paraguay, Argentina, and Brazil [1] and their genetic and behavioral characteristics have been well described [18–21]. However, studies on the biological activity of propolis in these species are scarce in the literature, particularly in view of the difficulty in finding colonies in their natural environment, where species are disappearing because of anthropogenic activity.

The pharmacological properties of propolis in these two species were evaluated by Velikova et al. [13], who described the antimicrobial activity of propolis extracts from* M. q. anthidioides*, and by Sawaya [10] who described the antioxidant activity of propolis extracts from* S. depilis*. In this context, the present study aimed to determine the chemical constituents as well as the antioxidant, cytotoxic, and toxic activities of ethanol extracts of propolis from the stingless bee species* S. depilis* and* M. q. anthidioides* from the state of Mato Grosso do Sul in Midwest Brazil.

## 2. Materials and Methods

### 2.1. Research Ethics

No specific permits were required for the described field studies. All field works to collect the propolis samples were conducted on private land and with owner permission. The field studies did not involve endangered or protected species. The protocol to collect of human peripheral blood was approved by the Research Ethics Committee (Comitê de Ética em Pesquisa; CEP) of the University Center of Grande Dourados (Centro Universitário da Grande Dourados; UNIGRAN), Brazil (CEP process number 123/12). All subjects provided written informed consent for participation.

### 2.2. Preparation of the Ethanol Extract of Propolis (EEPs)

Propolis samples from* S. depilis* (83.81 g) and* M. q. anthidioides* (36.42 g) were collected from the state of Mato Grosso do Sul (22°13′12′′S–54°49′2′′W), in the Midwest Region of Brazil, with a total of seven collections being performed for each species. The ethanol extract of propolis (EEPs) was prepared using 4.5 mL of 80% ethanol per 1 g of propolis. This mixture was incubated in a water bath at 70°C in a sealed container until total dissolution and subsequently filtered in filter paper qualitative 80 g/m^2^ (Prolab, São Paulo, Brazil) to obtain the EEPs of* S. depilis* (EEP-S) and* M. q. anthidioides* (EEP-M) [22]. After preparation of the extracts, they were kept at a temperature of −20°C until analysis.

### 2.3. Chemical Analysis

#### 2.3.1. Preparation of the Samples

The samples (1 mg) was fractionated with hexane and water in proportion 1 : 1 v : v and fraction soluble in hexane was analyzed by GC-MS and fraction in water by HPLC.

#### 2.3.2. GC-MS

Samples were injected and analyzed by gas chromatography-mass spectrometry (GC-MS). The GC-MS analysis was performed on a gas chromatograph (GC-2010 Plus Shimadzu Kyoto Japan) equipped with a mass spectrometer detector (GC-MS Ultra 2010) using LM-5 (5% phenyl dimethyl poly siloxane) capillary column (15 m length × 0.2 mm i.d. and 0.2 *μ*m film thickness) with initial oven temperature set at 150°C and heating from 150°C to 280°C at 15°C min^−1^ and a hold at 280°C for 15 min. The carrier gas was helium (99.99%) supplied at a flow rate of 1.0 mL/min, with split ratio 1 : 20, 1 *μ*L injection volume. The injector temperature was 280°C and the quadrupole detector temperature was 280°C. The MS scan parameters included an electron-impact ionization voltage of 70 eV mass range of 45–600 *m*/*z* and scan interval of 0.3 s. The identifications were completed by comparing the mass spectra obtained in the NIST21 and WILEY229 libraries. In some cases, the compound was confirmed by comparison of standards. Standards of the stigmasterol, *β*-sitosterol, *β*-amyrin, *α*-amyrin, *β*-amyrin acetate, and tocopherol (Sigma-Aldrich with purity ≥97%) were prepared in the concentration initial of 1000 *µ*g/mL. The concentrations of compounds were determined by extern calibration after dilutions appropriated in the range of 0.1–50 *µ*g/mL. The quantification of taraxasterol was performed in relation to stigmasterol. The procedure was performed in triplicate.

#### 2.3.3. HPLC

The extracts were analyzed in an analytical HPLC (LC-6AD, Shimadzu, Kyoto, Japan) system with a diode array detector (DAD) monitored at *λ* = 200–600 nm. The HPLC column was a C-18 (25 cm × 4.6 mm; particle size, 5 *μ*m; Luna, Phenomenex, Torrance, CA, USA), with a small precolumn (2.5 cm × 3 mm) containing the same packing, used to protect the analytical column. In each analysis, the flow rate and the injected volume were set as 1.0 mL min^−1^ and 20 *μ*L, respectively. All chromatographic analyses were performed at 22°C. Elution was carried out using an binary mobile phase of water with 6% acetic acid and 2 mM sodium acetate (eluent A) and acetonitrile (eluent B). The following applied gradients are as follows: 5% B (0 min), 15% B (30 min), 50% B (35 min), and 100% B (45 min). Standards of the vanilic acid, caffeic acid, ferulic acid, p-coumaric acid, benzoic acid, cinnamic acid, quercetin, luteolin, apigenin, and vanillin (Sigma-Aldrich, ≥97%) were prepared in the concentration initial of 1000 *µ*g/mL. The concentrations of compounds were determined by extern calibration after dilutions appropriated in the range of 0.01–10 *µ*g/mL. The procedure was performed in triplicate.

### 2.4. Antioxidant Activity

#### 2.4.1. DPPH Free Radical Scavenging Activity

The free radical-scavenger activity was determined by the DPPH (2,2-diphenyl-1-picrylhydrazyl) assay, as described previously by D. Gupta and R. K. Gupta [23] with some modifications. The antiradical activity of extracts was evaluated using a dilution series, in order to obtain a large spectrum of sample concentrations. This involved the mixing of 1.8 mL of DPPH solution (0.11 mM DPPH in 80% ethanol) with 0.2 mL of EEP-S or EEP-M (1–300 *μ*g/mL), followed by homogenization. After 30 min, quantification of the remaining DPPH radicals was recorded by using absorption set at 517 nm. Ascorbic acid and butylated hydroxytoluene (BHT) were used as reference antioxidants. The tests were performed in duplicate in 2 independent experiments. DPPH solution without the tested sample was used as control. The percentage inhibition was calculated from the control with the following equation:

(1) $$\begin{array}{c} \text{Scavenging activity }\left(\;\%\;\right)=\left(\;1-\frac{\text{Abs sample}}{\text{Abs control}}\;\right)\times 100. \end{array}$$

#### 2.4.2. ABTS Free Radical Scavenging Activity

Free radical scavenging capacity for EEP was studied as described by Re et al. [24], through the evaluation of the free radical scavenging effect on 2,2′-azinobis-(3-ethylbenzothiazoline-6-sulfonic acid) (ABTS) radical. The stock solutions included 7 mM ABTS solution and 140 mM potassium persulfate solution. The ABTS^•+^ radical was then prepared by mixing the two stock solutions (5 mL of ABTS solution and 88 *µ*L potassium persulfate solution) and left for 12–16 h at room temperature in the dark. The solution was then diluted by mixing 1 mL ABTS^•+^ radical with ethanol absolute to obtain an absorbance of 0.70 nm ± 0.05 units at 734 nm using a spectrophotometer. Then, 20 *µ*L of EEP-S or EEP-M (1–300 *µ*g/mL) was mixed with 1980 *µ*L of the ABTS^•+^ radical and the absorbance was taken at 734 nm after 6 min using a spectrophotometer. Ascorbic acid and butylated hydroxytoluene (BHT) were used as positive controls. Three independent experiments were performed in triplicate. The percentage of inhibition of the ABTS radical was calculated according to the following equation, where Abs_control_ is the absorbance of ABTS^•+^ radical without the tested sample:

(2) %  inhibition  of  ABTS=Abscontrol−AbssampleAbscontrol×100.

#### 2.4.3. Antioxidant Assay Using the Human Erythrocyte Model

*(1) Preparation of Erythrocyte Suspensions*. Following approval by the Research Ethics Committee, 20 mL of peripheral blood was collected from healthy donors into sodium citrate-containing tubes and was subsequently centrifuged at 1500 rpm for 10 min. After centrifugation, the blood plasma and leukocyte layers were discarded, and the erythrocytes were washed 3 times with saline solution and centrifuged at 1500 rpm for 10 min. Finally, 10% erythrocyte suspensions were prepared in saline solution to obtain 2.5% after the treatment.

*(2) Oxidative Hemolysis Inhibition Assay*. The protective effect of the propolis extracts was evaluated according to the method described by Campos et al. [12], with minor modifications. The assays were conducted with erythrocyte suspensions. The erythrocytes were preincubated at 37°C for 30 min in the presence of different concentrations of EEP or ascorbic acid (50–125 *µ*g/mL). Then, 50 mM 2,2′-azobis-(2-amidinopropane) dihydrochloride (AAPH) solution was added. A sample of 1 % ethanol was used as a negative control. Total hemolysis was induced by incubating erythrocytes with distilled water. Basal hemolysis caused by EEP was assessed by incubating erythrocytes with the extract without the presence of AAPH, and the control was assessed in erythrocytes incubated only with 0.9% NaCl. This mixture was incubated at 37°C for 240 min, with periodical stirring. Hemolysis was determined after every 120, 180, and of 240 minutes of incubation; specifically, sample were centrifuged at 1500 rpm for 10 min and aliquots of there were transferred to tubes with saline, after which the absorbance of the supernatant was read spectrophotometrically at 540 nm. The percentage hemolysis was measured with the formula *A*/*B* × 100, where (*A*) is the sample absorbance and (*B*) is the total hemolysis. Five independent experiments were performed in duplicate.

*(3) Inhibitory Efficiency against Lipid Peroxidation*. A 2.5% erythrocyte suspension was used to assess the protective effects of EEP against lipid peroxidation as described by Campos et al. [12] with some modifications. Erythrocytes were preincubated at 37°C for 30 min with different concentrations of EEPs or ascorbic acid (50–125 *μ*g/mL). A sample of 1% ethanol was used as a negative control. Next, 50 mM AAPH was added to the erythrocyte solution, which was then incubated at 37°C for 4 hours with periodical stirring. At 120, 180, and 240 minutes of incubation, the samples were centrifuged at 1500 rpm for 10 min, and 500 *μ*L aliquots of the supernatant were transferred to tubes with 1 mL of 10 nmol thiobarbituric acid (TBA). As a standard control, 500 *μ*L of 20 mM malondialdehyde (MDA) solution was added to 1 mL of TBA. The samples were incubated at 96°C for 45 min. The samples were then cooled, 4 mL of n-butyl alcohol was added and the samples were centrifuged at 3000 rpm for 5 min. The absorbance of supernatants sample was read at 532 nm. Three independent experiments were performed in duplicate. MDA levels in the samples were expressed in nmol/mL, obtained with the following formula:

(3) $$\begin{array}{c} \text{MDA}=\text{Abs sample}\times \left(\;20\times \frac{220.32}{\text{Abs standard}}\;\right). \end{array}$$

### 2.5. Cytotoxic Activity and Cell Death Profile

K562 erythroleukemia cells line was grown is suspension in RPMI 1640 media (Cultilab, Campinas, São Paulo, Brazil) supplemented with 10% fetal bovine serum (FBS; Cultilab), 100 U/mL of penicillin, and 100 *µ*g/mL of streptomycin in a humidified atmosphere at 37°C in 5% CO_2_. The cytotoxic activity and cell death profile were evaluated according to the method described by Paredes-Gamero et al. [25], with minor modifications. Cells were seeded into 96-well plates (2 × 10^4^ cell/well) and cultured in medium with 10% FBS in the absence or presence of EEP-S or EEP-M (31–500 *µ*g/mL) for 24 h. As negative controls were used cells were incubated with 0.2% ethanol (highest concentration of ethanol in extract). All effects of the EEPs were compared with negative controls. After this period, the K562 cells were washed with PBS and resuspended in annexin-labeling buffer (0.01 M HEPES, pH 7.4, 0.14 M NaCl, and 2.5 mM CaCl_2_). The suspensions were stained with annexin-FITC and propidium iodide (PI) (Becton Dickinson, Franklin Lakes, NJ, USA), according to the manufacture's instructions. The cells were incubated at room temperature for 15 min. Three thousand events were collected per sample, and the analyses were performed on a FACSCalibur flow cytometer (Becton Dickinson) with CellQuest software (Becton Dickinson).

### 2.6. In Vivo Toxicity

#### 2.6.1. Rearing and Maintenance of* Caenorhabditis elegans*

To perform the in vivo toxicity assay, we used the wild-type N2 strain of the nematode* Caenorhabditis elegans*. The specimens were incubated at 20°C in Petri dishes containing nematode growth medium (NGM) agar and fed with* Escherichia coli* strain OP50-1. The nematode culture was synchronized through treatment of pregnant hermaphrodites with 2% sodium hypochlorite and 5 M sodium hydroxide.

#### 2.6.2. Assessment of Toxicity in* C. elegans*

A toxicity assay for the EEPs was performed in* C. elegans* [26] in 96-well plates. Each well contained 10 nematodes at the L4 stage, which were incubated for 24 hours at 20°C with EEP-S and EEP-M at different concentrations (250–1000 *µ*g/mL) in M9 medium. After this period, nematode viability was evaluated by repeatedly touching the worms with a microspatula. For the manipulation and examination of nematodes, a model Motic SMZ-140 & W10X/23 (British Columbia, Canada) stereomicroscope was used. The data were calculated from two independent experiments in duplicate.

### 2.7. Statistical Analyses

All data are shown as the mean ± standard error of mean (SEM) and for statistical significant differences between the groups, using the analysis of variance (ANOVA) and posttest Dunnett, comparing the treatment with the control, using the Prism 6 GraphPad software. The results were considered significant when *p* < 0.05.

## 3. Results

### 3.1. Chemical Composition

The compounds identified in EEP-S and EEP-M are shown in Tables 1 and 2. Phytosterols, terpenes, phenolic compounds, and tocopherol were identified in the two extracts in different concentrations. EEP-S presented a higher content of amyrins (triterpenes) and *β*-sitosterol (phytosterols), whereas EEP-M exhibited a higher concentrations of tocopherol, amyrins, and apigenin (flavonoid). The compounds stigmasterol, taraxasterol, vanilic acid, caffeic acid, quercetin, luteolin, and apigenin were found only in EEP-M.

### 3.2. Antioxidant Activity

#### 3.2.1. DPPH and ABTS Free Radical Scavenging Activity

EEP-S and EEP-M were observed to scavenge free radicals in vitro. In both of the evaluated methods, EEP-M showed better antioxidant activity compared with EEP-S. In the DPPH assay, EEP-M showed 50% inhibition of free radicals (IC_50_) at a concentration of 60.91 ± 2.01 *µ*g/mL. The IC_50_ was not calculated for EEP-S. The maximum activity of EEP-M was achieved at a concentration of 300 *µ*g/mL (Table 3).

In the assay with the ABTS radical, IC_50_ values of the EEPs were 80.04 ± 0.31 *µ*g/mL (EEP-S) and 13.45 ± 1.81 *µ*g/mL (EEP-M), and they showed maximal activity at concentrations of 200 *µ*g/mL and 100 *µ*g/mL, respectively. The antioxidant activity of EEP-M was similar to that of the synthetic antioxidant BHT (Table 3).

#### 3.2.2. Oxidative Hemolysis Inhibition Assay

**Figure 1:**
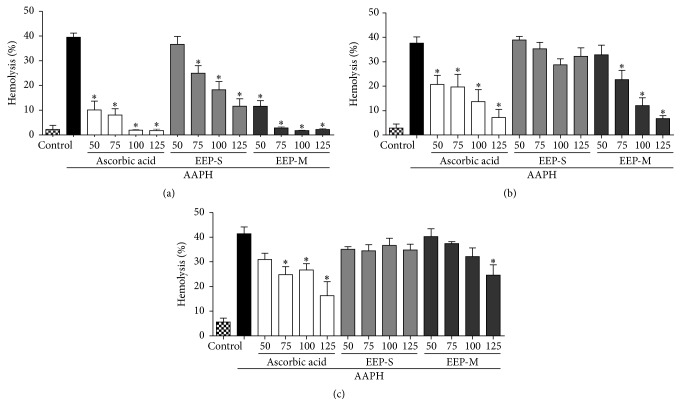
Protective effects of ascorbic acid (standard antioxidant) and ethanol extracts of propolis from* S. depilis* and* M. q. anthidioides* against AAPH-induced hemolysis determined using a human erythrocyte suspension at 120 min (a), 180 min (b), and 240 min (c). Ethanol was employed as a negative control. The results are expressed as the mean ± SEM (standard error of the mean), *n* = 5. ^*∗*^Significantly different (*p* < 0.05) compared with the AAPH group.

The standard antioxidant ascorbic acid and the EEPs showed concentration- and time-dependent antihemolytic activity. EEP-S decreased hemolysis for 120 min, with hemolysis inhibition reaching 63.5 ± 10.7% at a concentration of 125 *µ*g/mL, when compared with the AAPH sample. At the same concentration, ascorbic acid and EEP-M protected erythrocytes against hemolysis induced by the oxidant 2,2′-azobis(2-aminopropane) hydrochloride (AAPH) for up to 240 min, with hemolysis inhibition reaching 56.5 ± 12.8% and 37.7 ± 10.4% at 240 min, respectively, compared with erythrocytes treated with AAPH (Figure 1). At the various concentrations tested, the basal hemolysis observed using ascorbic acid and EEPs without the AAPH inducer was similar to the control treatments with saline and ethanol (data not shown).

#### 3.2.3. Efficiency of EEPs in the Inhibition of AAPH-Induced Lipid Peroxidation

**Figure 2:**
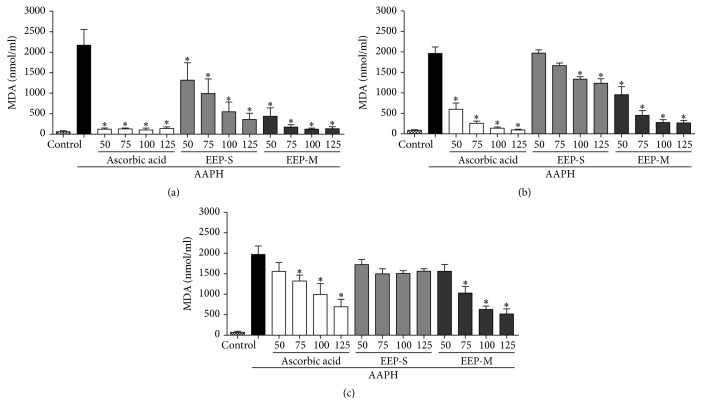
Protective effects of ascorbic acid (standard antioxidant) and ethanol extracts of propolis from* S. depilis* and* M. q. anthidioides* against the production of malondialdehyde (MDA)–a byproduct of lipid peroxidation–in a human erythrocyte suspension at 120 min (a), 180 min (b), and 240 min (c). Ethanol was used as a negative control. The results are expressed as the mean ± SEM (standard error of the mean), *n* = 3. ^*∗*^Significantly different (*p* < 0.05) compared with the AAPH group.

The effectiveness of EEPs in inhibiting lipid peroxidation induced by AAPH in human erythrocytes can be determined by measuring malondialdehyde (MDA) levels. Ascorbic acid and EEPs decreased MDA levels in a concentration- and time-dependent manner. EEP-S inhibited lipid peroxidation for 180 min. The ascorbic acid control solution inhibited lipid peroxidation by 65.6 ± 8.9%, whereas EEP-M inhibited peroxidation by 74.4 ± 6.1% for 240 min at a concentration of 125 *µ*g/mL, when compared with the AAPH sample (Figure 2).

### 3.3. Cytotoxic Activity and Cell Death Profile

**Figure 3:**
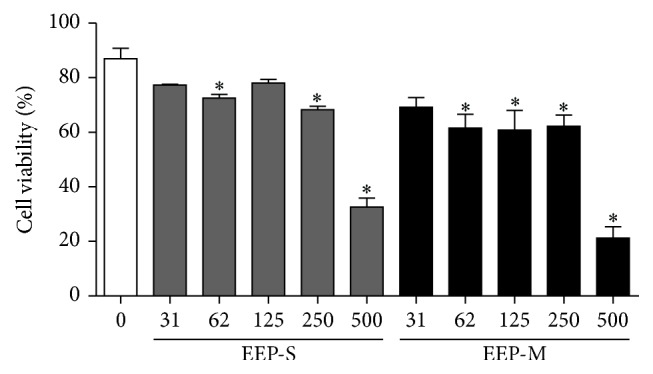
Cytotoxic activity of EEPs from* S. depilis* (EEP-S) and* M. q. anthidioides* (EEP-M) against the K562 erythroleukemia cell line. ^*∗*^*p* < 0.05 for the treated group versus control viable cells.

**Figure 4:**
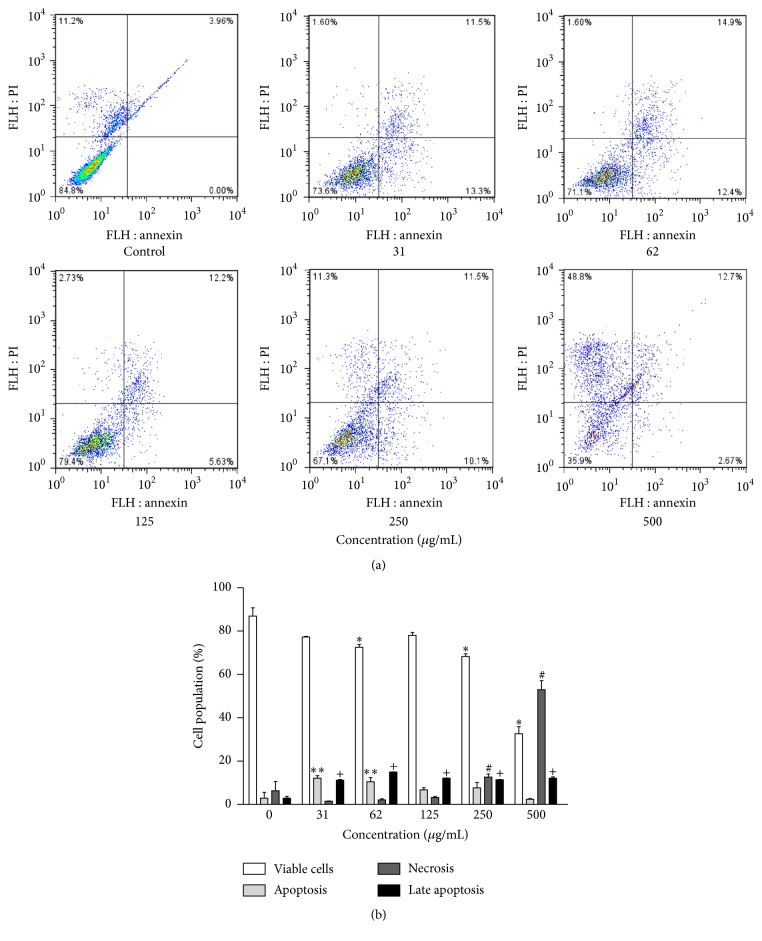
Cytotoxic action of EEP from* S. depilis* against the K562 erythroleukemia cell line. (a) Representative diagrams obtained via flow cytometry of cells stained with annexin V-FITC/PI: the lower left quadrant (PI−/An−) represents viable cells; the lower right quadrant (PI−/An+) represents apoptotic cells; the upper left quadrant (PI+/An−) represents cells undergoing necrosis; and the upper right quadrant (PI+/An+) represents cells in late apoptosis. (b) Frequency of cell death, obtained from the corresponding diagrams for the tested concentrations. ^*∗*^*p* < 0.05 for the treated group versus control viable cells. ^*∗∗*^*p* < 0.05 for the treated group versus control apoptosis. ^#^*p* < 0.05 for the treated group versus control necrosis. ^+^*p* < 0.05 for the treated group versus control late apoptosis.

**Figure 5:**
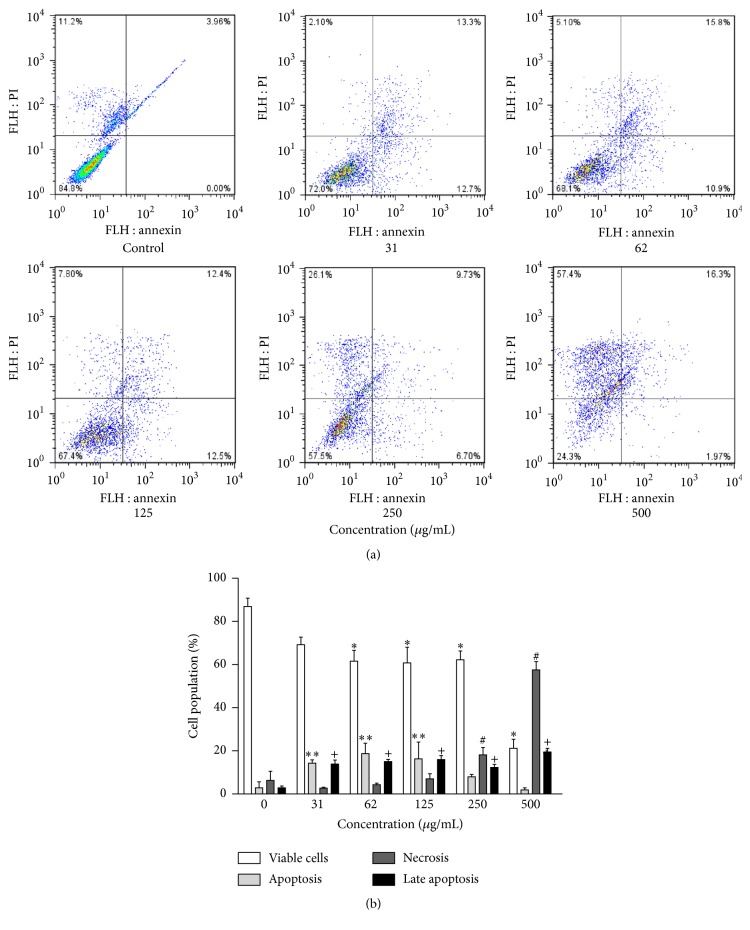
Cytotoxic action of EEP from* M. q. anthidioides* against the K562 erythroleukemia cell line. (a) Representative diagrams obtained via flow cytometry of cells stained with annexin V-FITC/PI: the lower left quadrant (PI−/An−) represents viable cells; the lower right quadrant (PI−/An+) represents apoptotic cells; the upper left quadrant (PI+/An−) represents cells in necrosis; and the upper right quadrant (PI+/An+) represents cells in late apoptosis. (b) Frequency of cell death, obtained from the corresponding diagrams for the tested concentrations. ^*∗*^*p* < 0.05 for the treated group versus control viable cells. ^*∗∗*^*p* < 0.05 for the treated group versus control apoptosis. ^#^*p* < 0.05 for the treated group versus control necrosis. ^+^*p* < 0.05 for the treated group versus control late apoptosis.

The ethanol extracts of propolis showed concentration-dependent cytotoxicity. At the highest concentration tested (500 *µ*g/mL), the cell growth of erythroleukemic cells (K562) were 32.6 ± 3.2% (EEP-S) and 21.2 ± 4.1% (EEP-M) (Figure 3). At this concentration, after 24 h of treatment, EEP-S promoted death by necrosis in 52.9 ± 4.1% of cells and death by late apoptosis in 12.1 ± 0.6% of cells (Figures 4(a) and 4(b)), whereas EEP-M promoted death by necrosis in 57.5 ± 3.8% of cells and death by late apoptosis in 19.4 ± 1.6% of cells (Figures 5(a) and 5(b)).

### 3.4. Toxicity in* C. elegans*

**Figure 6:**
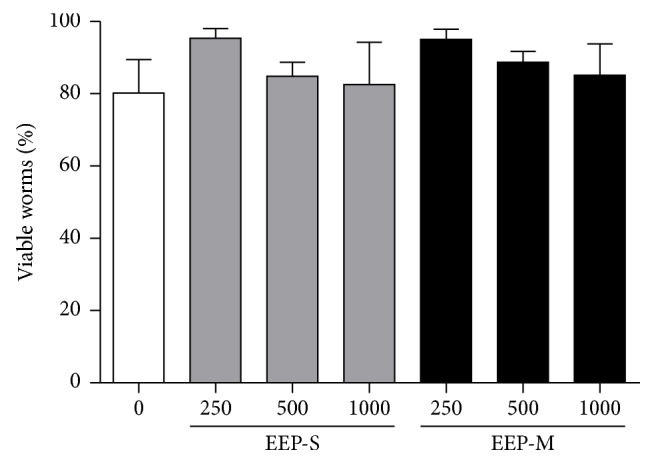
Toxicity of EEPs from* S. depilis* EEP-S and* M. q. anthidioides* (EEP-M) against* C. elegans*. ^*∗*^*p* < 0.05 for the treated group versus the control with untreated nematodes.

EEP-S and EEP-M were not toxic to the nematodes after 24 h of incubation at any of the concentrations evaluated compared with the control group (Figure 6).

## 4. Discussion

Propolis is a bee product that is widely used in the cosmetics and food industries and is considered a functional food (nutraceutical) able to prevent diseases when included in food products [5]. The chemical constituents present in propolis are responsible for its therapeutic properties [7, 11, 27], including its antibacterial, antifungal, and antiviral activities [5, 14] as well as its anti-inflammatory and antitumor activities [15, 16, 28, 29].

The major compounds identified in the EEP-S were *β*-amyrin, *β*-amyrin acetate, and *α*-amyrin and in the EEP-M were tocopherol, *β*-amyrin acetate, and apigenin. Both extracts show similar amounts of *β*-amyrin, vanillin, p-coumaric acid, ferulic acid, cinnamic acid, and benzoic acid; however, the EEP-S showed higher content of amyrins than EEP-M. By contrast, EEP-M exhibited approximately four times the amount of tocopherol found in EEP-S and other compounds which were found exclusively on the EEP-M. Despite presenting the same chemical constituents, variations in the concentrations of these compounds may influence the biological activities of the extracts.

The compounds phenolic and flavonoid are correlated with the antioxidant and antitumor activity of propolis [8, 10–12, 30]. Additionally, other compounds identified in the propolis such as caffeic acid, apigenin, and triterpenes are descript with important blockers of oncogenic kinase PAK1, well known to be responsible for a variety of diseases such as infectious diseases, Alzheimer's disease, diseases inflammatory, diabetes, hypertension, obesity, and cancer [31].

Phenolic compounds and terpenes have been found in propolis extracts of other species of stingless bees from the same geographical region [12, 29], which may be related to the plant species from which the bees collect raw materials for propolis production.

The terpenes and phenolic compounds found in EEPs have been described as compounds responsible for the antioxidant activities of various plant species [32–34]. Antioxidants are compounds that, when present at low concentrations, retard or prevent the oxidation of substrates and are highly beneficial to health due to protecting cells and macromolecules from oxidizing agents [35].

The most common oxidants in the body include the superoxide (O_2_^−^), hydroxyl (OH^•^), peroxyl (ROO), alkoxyl (RO), and hydroperoxyl (HO_2_) radicals, which are collectively known as reactive oxygen species (ROS). These free radicals are produced via gradual reduction of molecular oxygen and generate unpaired electrons, which cause oxidative stress when they are out of equilibrium [36].

Both EEPs stabilized the free radicals 2,2-diphenyl-1-picrylhydrazyl (DPPH) and 2,2′-azino-bis(3-ethylbenzothiazoline-6-sulfonic acid) (ABTS). However, EEP-M showed higher antioxidant activity than EEP-S, which may be related to the different concentrations of tocopherol in the extracts. Some studies have reported the importance of tocopherols as antioxidants [37, 38].

In addition, the amyrins may be associated with the antioxidant activity of the extracts. Tocopherols and these triterpenes are fat-soluble antioxidants that scavenge ROS [34, 39, 40]. These compounds may have been responsible for the increased antioxidant activity of the EEPs observed in the assay with the free radical ABTS, as this method is applied to hydrophilic and lipophilic antioxidant systems [41]. Therefore, the higher solubility of these compounds in the solvent used in this assay produced greater antioxidant activity.

These results corroborate those obtained in the assays involving the inhibition of lipid peroxidation, in which the EEPs presented antihemolytic activity and protective activity against lipid peroxidation when incubated with human erythrocytes in the presence of an oxidizing agent. EEPs may also inhibit the peroxyl radical (ROO), which induces peroxidation of lipids and proteins present in human erythrocyte membranes [42].

Oxidative stress leads to lipid peroxidation and, consequently, cell damage due to the oxidation of essential cellular compounds, including lipids, proteins, and nucleic acids. An excess of these free radicals can promote cell aging and the development of various diseases, including Alzheimer's, cancer, arthritis, and diabetes, and can increase the risk of cardiovascular disease [36, 43].

Therefore, the evaluated EEPs contain important antioxidant compounds that can limit the spread of oxidative stress-related diseases. The free radicals scavenging and antihemolytic ability demonstrated by the EEP-M were more efficient than results observed for propolis from the stingless bee* Tetragonisca fiebrigi* [29] and* Melipona orbignyi* [12] from Midwest Region of Brazil and some extracts of* Apis mellifera* [44, 45].

In the present study, EEP-S and EEP-M exhibited cytotoxic activity against K562 erythroleukemic cells. In addition, the decrease in cell viability was greater in cells treated with EEP-M than in those treated with EEP-S. However, both EEPs caused necrosis in most of the cells at a concentration of 500 *µ*g/mL. The cytotoxic effect of propolis was also observed in other cell lines as human lung adenocarcinoma epithelial (A549), human cervical adenocarcinoma (HeLa), and human breast adenocarcinoma (MCF-7) but the mechanisms involved in the death of these tumor cells were apoptosis [46–48]. Therefore, the use of EEP-S and EEP-M may constitute an alternative treatment for chronic myeloid leukemia, as K562 cells are resistant to apoptosis induced by various agents [49].

Some compounds found in EEPs may play an important role in anticancer activity, including tocopherol, which shows antitumor activity in esophageal cancer cells [50] and breast cancer in vitro and in vivo [51]. Furthermore, caffeate derivatives are cytotoxic against human carcinoma cell lines [52].

Other phenolic compounds present in propolis exhibit antiproliferative and cytotoxic effects against various tumor cell lines, including those obtained from renal cell carcinomas [53] and the colon [30], pancreas [54], skin [55], and lungs [56]. Amyrins can be isolated from plants and is known as natural potent anticancer; its compounds induces tumor cell death as human bladder carcinoma (NTUB1) [34, 57] and leukemia cells (HL-60) [58].

Although the EEPs presented cytotoxic activity against K562 cells, no toxic or lethal effects were observed against the nematode* C. elegans*.

In vivo experimental models serve as a tool to understand effects of natural products in whole organisms. These results suggest that the evaluated propolis samples show specificity against leukemic cells, considering that these nematodes were not affected. This specificity may be important for the treatment of leukemia because drug toxicity and low specificity are among the major difficulties in the treatment of this disease [37].

Corroborating with the toxicity data of the EEPs, recent study showed that the crude extract of propolis presented anticancer effects in human lung cancer cell and is antimelanogenic in the melanoma cell line; additionally it was able to prolong the life of* C. elegans* [48]. In addition, the caffeic acid, the major constituent of propolis, does no present toxic effects and also was able to increase the survival of the nematode* C. elegans* after infection with the fungal pathogen [59]. The ability of propolis or caffeic acid to extend lifespan in* C. elegans* was associated with inactivation of oncogenic kinase PAK1 [48, 59].

Previous studies have shown that* C. elegans* can be used as an experimental model for obtaining rapid results in toxicity studies for pharmacological compounds [60, 61] because it is a multicellular organism with a high reproduction rate and short life cycle, which makes it an excellent in vivo model for complementing cell culture-based systems [61].

Therefore, we conclude that the tested EEPs exhibit antioxidant and cytotoxic activities, attributed to their chemical composition, which includes phytosterols, terpenes, phenolic compounds, and tocopherol, and possibly to the synergy between different compounds present in propolis. Moreover, these EEPs show therapeutic potential for use in the prevention and treatment of diseases associated with oxidative stress and the proliferation of tumor cells.

## Figures and Tables

**Table 1:** Compounds identified in unpolar fraction of the EEPs from *Scaptotrigona depilis* and *Melipona quadrifasciata anthidioides* by GC-MS.

Peak	Retention time (min)	Compounds	Molecular mass	EEP-S (mg/g)	EEP-M (mg/g)
1	17.02	Stigmasterol^*∗*^	412	—	4.8 ± 0.1
2	17.72	*β*-Sitosterol^*∗*^	414	9.6 ± 0.2	5.4 ± 0.2
3	17.93	*β*-Amyrin^*∗*^	426	14.3 ± 0.3	11.6 ± 0.3
4	18.09	Taraxasterol	426	—	3.0 ± 0.1
5	18.45	*α*-Amyrin^*∗*^	426	10.5 ± 0.3	5.0 ± 0.1
6	19.65	*β*-Amyrin acetate^*∗*^	468	21.5 ± 0.4	13.7 ± 0.4
7	24.56	Tocopherol^*∗*^	430	3.6 ± 0.1	15.0 ± 0.5

^*∗*^Compound was confirmed by comparison of standard.

Data are shown as media ± standard deviation.

**Table 2:** Compounds identified in polar fraction of the EEPs from *Scaptotrigona depilis* and *Melipona quadrifasciata anthidioides * by HPLC.

Peak	Retention time (min)	Compounds	Molecular mass	EEP-S (mg/g)	EEP-M (mg/g)
1	7.95	Vanilic acid	168	—	5.9 ± 0.1
2	8.64	Caffeic acid	180	—	6.1 ± 0.2
3	10.44	Vanillin	152	5.5 ± 0.2	5.7 ± 0.1
4	13.48	p-Coumaric acid	164	6.3 ± 0.2	6.1 ± 0.2
5	17.28	Ferulic acid	194	5.4 ± 0.2	6.1 ± 0.2
6	19.99	Benzoic acid	122	6.8 ± 0.2	6.9 ± 0.1
7	35.33	Quercetin	302	—	9.9 ± 0.2
8	36.68	Luteolin	286	—	1.3 ± 0.1
9	40.01	Cinnamic acid	148	13.4 ± 0.4	13.2 ± 0.3
10	42.62	Apigenin	270	—	15.6 ± 0.4

^*∗*^Compound was confirmed by comparison of standard.

Data are shown as media ± standard deviation.

**Table 3:** IC_50_ and maximum DPPH and ABTS radical scavenging activity of standard antioxidants, EEP-S and EEP-M.

Sample	DPPH			ABTS		
	IC_50_ (*µ*g/mL)	Maximum inhibition		IC_50_ (*µ*g/mL)	Maximum inhibition	
		%	*µ*g/mL		%	*µ*g/mL
Ascorbic acid	3.32 ± 0.65	96.75 ± 0.41	50	2.50 ± 0.48	97.37 ± 1.55	10
BHT	22.84 ± 7.87	89.36 ± 2.30	200	20.46 ± 2.78	95.36 ± 1.80	100
EEP-S	ND	14.91 ± 1.73	300	80.04 ± 0.31	73.42 ± 3.47	200
EEP-M	60.91 ± 2.01	97.47 ± 0.03	300	13.45 ± 1.81	99.31 ± 0.12	100

Values are means ± SEM. DPPH (*n* = 2) and ABTS (*n* = 3). ND: not determined.

## Abbreviations

AAPH: 2,2′-Azobis-(2-amidinopropane) dihydrochloride
Abs: Absorbance
An: Annexin V-FITC
BHT: Butylhydroxytoluene
DAD: Diode array detector
DPPH: 2,2-Diphenyl-1-picrylhydrazyl
CG-MS: Gas chromatography-mass spectrometry
HPLC: High performance liquid chromatography
EEPs: Ethanol extract of propolis
EEP-S: Ethanol extract of propolis of* Scaptotrigona depilis*
EEP-M: Ethanol extract of propolis of* Melipona quadrifasciata anthidioides*
MDA: Malondialdehyde
NaCl: Sodium chloride
PI: Propidium iodide
SEM: Standard error of the mean
TBA: Thiobarbituric acid.
